# Homer1a attenuates glutamate-induced oxidative injury in HT-22 cells through regulation of store-operated calcium entry

**DOI:** 10.1038/srep33975

**Published:** 2016-09-29

**Authors:** Wei Rao, Cheng Peng, Lei Zhang, Ning Su, Kai Wang, Hao Hui, Shu-hui Dai, Yue-fan Yang, Peng Luo, Zhou Fei

**Affiliations:** 1 Department of Neurosurgery, Xijing Hospital, Fourth Military Medical University, Xi’an, 710032, P. R. China; 2 Department of Radiotherapy, Xijing Hospital, Fourth Military Medical University, Xi’an, 710032, P. R. China

## Abstract

Calcium disequilibrium is extensively involved in oxidative stress-induced neuronal injury. Although Homer1a is known to regulate several neuronal calcium pathways, its effects on, or its exact relationship with, oxidative stress-induced neuronal injury has not yet been fully elucidated. We found that Homer1a protected HT-22 cells from glutamate-induced oxidative stress injury by inhibiting final-phase intracellular calcium overload and mitochondrial oxidative stress. In these cells, stromal interactive molecule 1 (STIM1) puncta, but not the protein level, was significantly increased after glutamate treatment. Store-operated calcium entry (SOCE) inhibitors and cells in which a key component of SOCE (STIM1) was knocked out were used as glutamate-induced oxidative stress injury models. Both models demonstrated significant improvement of HT-22 cell survival after glutamate treatment. Additionally, increased Homer1a protein levels significantly inhibited SOCE and decreased the association of STIM1-Orai1 triggered by glutamate. These results suggest that up-regulation of Homer1a can protect HT-22 cells from glutamate-induced oxidative injury by disrupting the STIM1-Oria1 association, and then by inhibiting the SOCE-mediated final-phrase calcium overload. Thus, regulation of Homer1a, either alone or in conjunction with SOCE inhibition, may serve as key therapeutic interventional targets for neurological diseases in which oxidative stress is involved in the etiology or progression of the disease.

Oxidative stress is a well-established and comprehensive injury mechanism in chronic and acute neurological diseases, including Parkinson’s disease^1^, Alzheimer’s disease^2^, and amyotrophic lateral sclerosis^3^, as well as traumatic brain injury and stroke^4^,^5^. Oxidative stress is caused by a disequilibrium of reactive oxygen species (ROS) production and clearance, which can lead to neuronal death.

HT-22 cells, an immortalized mouse hippocampal cell line, are a good model of neuronal oxidative stress, as they lack ionotropic glutamate receptors (iGluRs). High doses of glutamate in these cells inhibit the cystine/glutamate exchanger, which leads to a reduction in glutathione production, and subsequently leads to an imbalance of intracellular ROS production and elimination. Ultimately, this process results in cell death by oxytosis^6^,^7^. This model of glutamate-induced HT-22 cell death has been extensively used as an endogenous oxidative stress model, and is the model we chose to investigate the mechanism involved in neuronal oxidative stress for these studies.

Previous studies have indicated that both an increase of ROS and strong calcium influx are important aspects of glutamate-induced HT-22 cell death^8^. Furthermore, inhibition of calcium influx with either the calcium channel blocker CoCl_2_, or the calcium chelator EGTA attenuated glutamate-induced HT-22 cell death^9^,^10^,^11^. Therefore, it is of crucial importance that we investigate the mechanism of glutamate-induced calcium dysregulation in HT-22 cells, as well as the related neuroprotection molecules.

One protein that may play an important role in glutamate-induced calcium dysregulation is Homer1a, an intensively-studied immediate early gene (IEG) that belongs to the postsynaptic protein family. Previous studies have demonstrated that Homer1a is extensively involved in neuronal calcium signals, affecting not only metabolic glutamate receptors (mGluR), but also iGluRs, particularly N-methyl-D-aspartate receptor^12^,^13^,^14^,^15^,^16^. These functions are mostly attributed to its structural features. Homer1a has an enabled/vasodilator-stimulated phosphoprotein (Ena/VASP) homology 1 (EVH1) domain, which is a conserved domain among all Homer proteins (including Homer1b/c and Homer2); however, it lacks the C-terminal coiled-coil (CC) domain involved in self-multimerization of the other Homer proteins^17^.

Accumulating evidence has indicated that Homer1b/c can regulate the active or inactive status of calcium channels and related regulating proteins, like mGluR5, transient receptor potential channels (TRPC), and L-type voltage-dependent calcium channel subunit α1c (Ca_V_1.2), by recognizing and binding to the PPXXF or LPSSP motif found in those proteins with its EVH1 domain, and then self-multimerizing with its CC domain^15^,^18^,^19^. Interestingly, Homer1b/c can also alter the store-operated calcium entry (SOCE)-mediated calcium influx through the interaction Homer1b/c with both of stromal interactive molecule 1 (STIM1) and the Ca^2+^ release-activated Ca^2+^ channel Orai1 in human platelets^20^. SOCE, which is mediated by sensor proteins, the stromal interactive molecules (STIM, mainly STIM1 and STIM2), and Ca^2+^ release-activated Ca^2+^ channels (mainly Orai1 and Orai2), plays a key role in maintaining intracellular calcium homeostasis in both excitable and non-excitable cells^21^.

Extensive studies have established the relationship between SOCE and oxidative stress^22^,^23^,^24^,^25^. Additionally, the inhibition of SOCE alleviated oxidative stress-induced cell injury in HT-22 cells by reducing calcium influx^8^,^26^. Because Homer1a lacks the CC domain, and therefore cannot self-multimerize, Homer1a might act as a negative competitor of Homer1b/c, disturbing the Homer1b/c-calcium channel complexes, and therefore play a role in the cell’s response to oxidative stress^12^,^13^,^27^. However, whether Homer1a plays a role in glutamate-induced HT-22 injury or can alter glutamate-induced calcium influx has not been elucidated.

In this study, we used a glutamate-induced endogenous oxidative stress model to explore the role and the possible interaction with SOCE of Homer1a in oxidative stress injury. The results uncovered a neuroprotective role of Homer1a in glutamate-induced final phase calcium overload and mitochondrial oxidative stress. Up-regulation of Homer1a protected HT-22 cells from glutamate-induced injury. This neuroprotection might partially be due to the role of Homer1a in dissociating STIM1-Orai1 association. Therefore, therapeutic strategies that may up-regulate Homer1a and/or inhibit SOCE might be an important intervention in neurological diseases in which oxidative stress is involved.

## Results

### Increased levels of Homer1a attenuated glutamate-induced HT-22 cell injury

**Figure 1:**
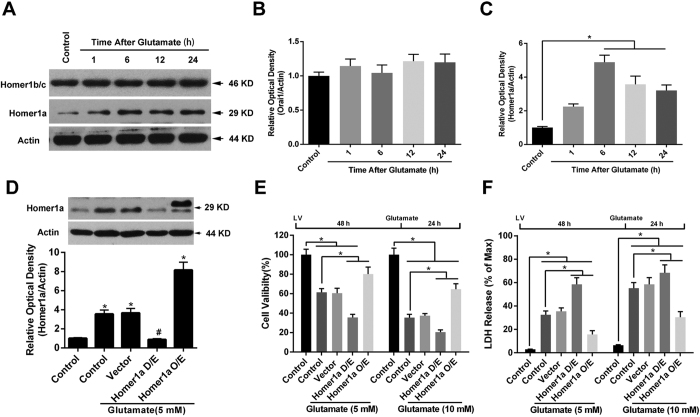
Increased Homer1a protein levels improved HT-22 cell survival after glutamate treatment. Twenty-four h after seeding the cells into 60-mm plates, the HT-22 cells were exposed to glutamate (10 mM) for either 1, 6, 12 or 24 h. The proteins levels of Homer1b/c and Homer1a were evaluated by western blotting (**A**). The relative expressions of Homer1b/c (**B**), and Homer1a (**C**) were calculated as percent of the optical density of the control group. After infection with either Homer1a shRNA or Homer1a O/E lentiviral vectors, the HT-22 cells were exposed to glutamate (5 mM) for 6 h, after which the proteins were harvested for western blotting assay (**D**). Forty-eight h after lentiviral infection, followed by 24 h of glutamate treatment, cellular viability was assessed by CCK-8 assay as a percentage of control group (**E**), and the cell injury was detected by LDH release assay (**F**). HT-22 cells in the control group were not subjected to lentiviral infection or glutamate injury. Data are presented as mean ± SEM from four experiments; *p < 0.05. ^#^p < 0.05 vs. control + glutamate or vector + glutamate group.

To test the role of Homer1a in glutamate-induced HT-22 injury, we first analyzed the protein levels of Homer1a and Homer1b/c at 1, 6, 12 and 24 h after glutamate treatment. As shown in Fig. 1A,B, the levels of Homer1b/c protein did not change after glutamate treatment. In contrast, the protein levels of Homer1a showed significant elevation at 6, 12 and 24 h after the glutamate treatment (Fig. 1A,C).

We then constructed lentivirus-based shRNA (D/E) and overexpression (O/E) vectors to regulate the protein levels of Homer1a. As shown in Fig. 1D, transfection of Homer1a D/E vectors for 48 h decreased Homer1a protein levels by approximately 75% compared with that of the control + glutamate and vector + glutamate groups 6 h after glutamate treatment. Furthermore, transfection of Homer1a O/E vectors for 48 h significantly up-regulated the Homer1a protein levels by approximately 3 fold as compared with that of the control + glutamate and vector + glutamate groups. Since no significant changes were observed between the Scr-shRNA group and the vector group, the Scr-shRNA group has not been shown in our experiments.

Consistent with previous studies, glutamate treatment caused a dose-dependent decrease in cell viability, with a 38.55% decrease in cell viability at 5 mM, and a 64.56% decrease at 10 mM (Fig. 1E). Cells that were not treated with glutamate were included as non-injured controls. Corresponding results were obtained with an LDH releasing assay (Fig. 1F). The decrease in Homer1a protein levels with the D/E vector significantly mitigated glutamate-induced HT-22 cell injury as evidenced by a further reduction in cell viability (Fig. 1E) and a further increase in LDH release (Fig. 1F) at both concentrations. In contrast, increased levels of Home1a protein from using the O/E vector significantly improved HT-22 cell survival and mitigated LDH release against glutamate treatment (Fig. 1E,F). Taken together, these results indicate that increased levels of Homer1a protein attenuated glutamate-induced HT-22 cells injury.

### Increased Homer1a protein levels attenuated glutamate-induced calcium overload and mitochondrial oxidative levels

**Figure 2:**
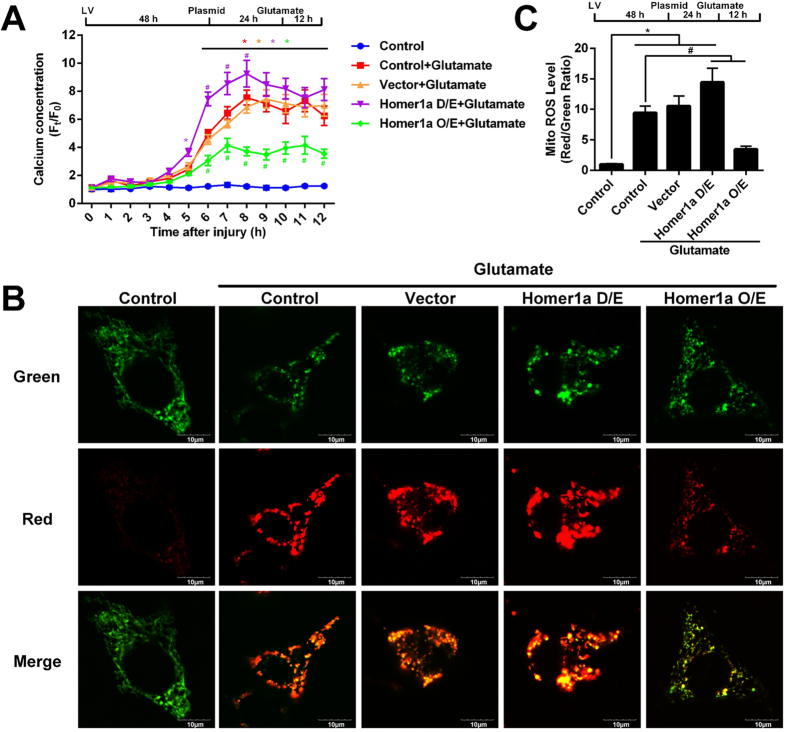
Increased Homer1a protein levels attenuated calcium overload and protected against mitochondrial damage after glutamate treatment. For long-term [Ca^2+^]^cyt^ monitoring, after infection with lentivirus particles, the HT-22 cells were transfected with genetically encoded Ca^2 +^ indicator construct (pCMV-B-GECO1) for 24 h. Then, the HT-22 cells were treated with glutamate, and the [Ca^2+^]_cyt_ was measured at intervals of 1 h for 12 h. The data are represented as time-series curve graph (**A**). The fluorescence changes were calculated as: total fluorescence value/total cell number. Cells in which the fluorescence signal quenched were excluded from the analysis. To visualize mitochondrial oxidative stress level, HT-22 cells were infected with lentiviral particles for 48 h, followed by transfection with pMitotimer. Twenty-four h after transfection, the HT-22 cells were treated with glutamate for 12 h, and imaged with confocal microscopy. Representative images of mitochondrial oxidative stress are shown (**B**, Green, non-oxidative levels; Red, oxidative levels), and mitochondrial oxidative levels were quantitated (**C**). Scale bar = 10 μm. Approximately 30–40 HT-22 cells were analyzed per group. HT-22 cells in the control group were not subjected to lentiviral infection or glutamate injury. Data are presented as mean ± SEM from four experiments. *p< 0.05 vs. control group; ^#^*p* < 0.05 vs. control + glutamate or vector + glutamate group.

Previous evidence has indicated that both intracellular ROS accumulation and Ca^2+^ overload were the main causes of glutamate-induced HT-22 cell injury. Therefore, to understand the mechanism of Homer1a-mediated neuroprotection, we measured intracellular calcium dynamics and mitochondrial oxidative levels using live-cell imaging of cells transfected with either a genetically encoded Ca^2+^ indicator construct (pCMV-B-GECO1) or a mitochondrial ROS reporter construct (pMitotimer). As shown in Fig. 2A, when compared with that of non-treated control group, glutamate treatment significantly increased intracellular calcium levels in the control + glutamate and vector + glutamate groups; however, this change was not apparent in the first 4 h after glutamate treatment. Homer1a O/E significantly attenuated glutamate-induced intracellular Ca^2+^ levels (Fig. 2A), as compared with that of the control + glutamate or the vector + glutamate groups. In contrast, decreased Homer1a protein levels using the D/E vector significantly increased intracellular Ca^2+^ levels at 6, 7, and 8 h after glutamate as compared to the control + glutamate or vector + glutamate groups.

**Figure 3:**
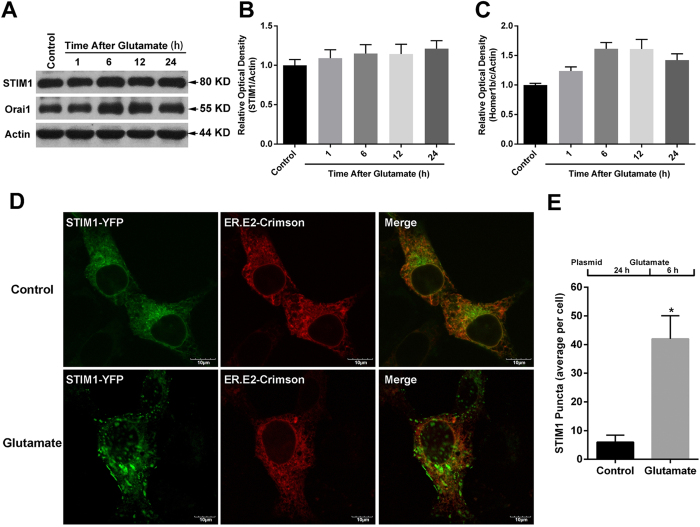
Glutamate induced the formation of STIM1 puncta in HT-22 cells. Twenty-four h after seeding the cells into 60-mm plates, the HT-22 cells were exposed to glutamate (10 mM) for either 1, 6, 12 or 24 h. The protein levels of STIM1 and Orai1 were evaluated by western blotting (**A**). The relative expressions of STIM1 (**B**) and Orai1 (**C**) were calculated as percent of the optical density of the control group. After transfection with STIM1-YFP and ER.E2-Crimson constructs for 24 h and then exposure to glutamate (10 mM) for 6 h, the STIM1-YFP distribution was assessed via confocal microscopy; representative images are shown (**D**). Scale bar = 10 μM. The average STIM1-YFP puncta per cell was calculated (**E**). Approximately 30 to 35 cells were analyzed in each experiment. Data are presented as mean ± SEM from four experiments; *p < 0.05 vs. control group.

Oxidative stress can also cause mitochondrial damage, so we next used fluorescent microscopy to examine the oxidative levels of the mitochondria in these cells. Under physiological conditions, mitochondria in the control group exhibited a low red/green ratio (Fig. 2B,C, column 1). However, 12 h after glutamate insult, the mitochondria in the control + glutamate and vector + glutamate groups appeared high oxidative stress in these cells as evidenced by high red/green ratio (Fig. 3B,C, Column 2 and 3). Decreased levels of Homer1a protein in the cells transfected with the D/E vector indicated a significant increase of oxidative levels as compared to the control + glutamate and vector + glutamate groups (Fig. 3B; evidenced by a higher red/green ratio). Additionally, increased Homer1a protein levels in the cells transfected with the O/E vector significantly alleviated the oxidative stress (Fig. 3B,C, Column 5) as compared to the control + glutamate and vector + glutamate groups. From these results, we conclude that Homer1a may protect against glutamate-induced HT-22 cell injury by restoring intracellular calcium homeostasis and reducing mitochondrial oxidative stress.

### Glutamate-induced HT-22 injury redistributed STIM1 into puncta within the cell

Since HT-22 cells lack iGluRs, SOCE might be the main source of calcium influx in these cells. Thus, we further evaluated the change of two components of SOCE, namely STIM1 and Orai1, in this cell model system. As shown in Fig. 3A,B, the levels of STIM1 protein did not change after glutamate treatment. Additionally, although the expression of Orai1 seemed to slightly increase 6 h after injury, these changes were not statistically significant (Fig. 3A,C).

Previous studies have indicated that the cellular distribution of SOCE components greatly affected SOCE function. Therefore, we visualized the change of STIM1 distribution by transfecting the STIM1-YFP and ER.E2-Crimson constructs. Forty-eight h after transfection, the cells were treated with glutamate, or not, for 6 h. As shown in the Fig. 3D, the STIM1-YFP showed a distributed dispersion pattern, with predominant localization in the ER under normal physiological conditions. However, at 6 h after the glutamate treatment, the STIM1-YFP was differently distributed and appeared as STIM1-YFP puncta, suggesting that glutamate promotes STIM1 oligomerization. Compared with the control group, the STIM1-YFP puncta were significantly increased at 6 h the after glutamate treatment (Fig. 3E). We also detected the distribution of Orai1 within the cell using an Orai1-YFP construct, finding that Orai1 primarily distributed on the cell membrane, and no apparent redistribution of Orai1 after glutamate treatment was observed (data not shown). These results led us to conclude that glutamate treatment induces a redistribution of STIM1 into puncta within the cell.

### Inhibition of SOCE mitigated glutamate-induced HT-22 cells injury

**Figure 4:**
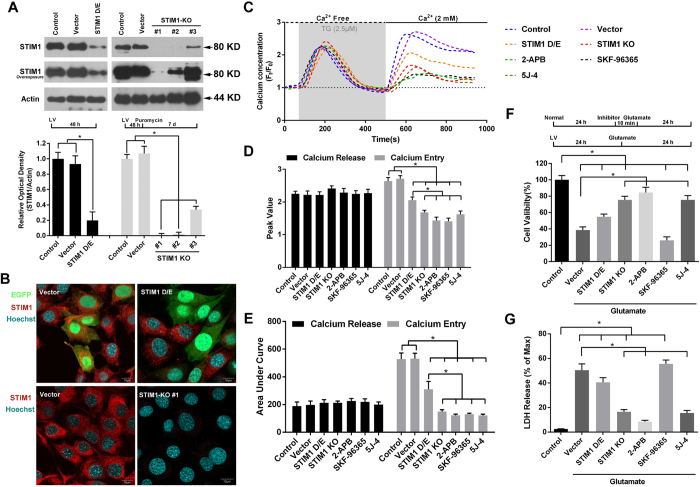
SOCE inhibition by knocking out of STIM1 or application of SOCE inhibitors attenuated glutamate-induced HT-22 cell injury. HT-22 cells were infected with lentivirus particles containing either scramble-shRNA (Scr-shRNA), or STIM1-shRNA for 48 h and harvested for western blotting assay (**A**, Left) to validate the efficiency of STIM1 down-regulation. For knockout of STIM1 expression, HT-22 cells were infected with CRISPR/Cas9-mediated particles with either vector or a STIM1 KO construct (#1, #2, and #3) for 48 h and then conditionally cultured with puromysin for 7 days. The expression of STIM1 was detected and represented as percent optical density of control cells (**A**, Right). STIM1 protein levels for cells treated with Scr-shRNA, STIM1-shRNA (MOI = 5), vector, or STIM1 KO #1 were also examined using immunofluorescence staining (**B**). After verification of the efficiency of STIM1 down-regulation or knockout, the HT-22 cells were loaded with Fura-red (4 μM) and baseline levels were recorded every 15 s for a total of 100 s. The HT-22 cells were then treated with 2.5 μM TG to release Ca^2+^ from calcium stores (gray zone) in Ca^2+^-free buffer (black dotted line) for 400 s. The buffer was then refreshed with Ca^2+^buffering (2 mM, black full line). Changes in Ca^2+^ signals were evaluated as time-series curve (**C**) and assessed as peak F/F_0_ (**D**) and area under curve (AUC, **E**). Approximately 40 to 50 cells were analyzed in each experiment. Twenty-four h after normal or lentivirus-infected HT-22 cells were seeded into 96-well or 6-well plates with identical cell density, the cells were pretreated with 2-APB (100 μM), SKF96365 (25 μM), or 5J-4 (20 μM) for 10 min, followed by treatment with glutamate. Cell viability (**F**) and LDH release (**G**) were assessed 24 h later. Data are presented as mean ± SEM from four experiments. *p < 0.05.

To further elucidate the role of SOCE in glutamate-induced HT-22 cells injury, we altered STIM1 levels using lentivirus-based STIM1-shRNA or STIM1-CRIPSR/Cas9 vectors to either down-regulate (D/E) or knockout (KO) the expression of STIM1, respectively. Consistent with our previous studies, STIM1-shRNA significantly decreased STIM1 protein levels by 80.27% as compared to the control group (Fig. 4A). Thirty-six h after transfection, at a low MOI of 5, the levels of STIM1 protein were notably decreased in EGFP-positive HT-22 cells transfected with STIM1-shRNA group as compared with that of EGFP-positive HT-22 cells transfected with scrambled shRNA (Scr-shRNA) or EGFP-negative non-transfected HT-22 cells (Fig. 4B, Row 1). Additionally, three different sgRNAs were used to KO the expression of STIM1. As shown in Fig. 3A, sgRNA #1 had the highest efficacy, with STIM1 protein levels almost undetectable even after overexposure of the western blot. Similar results were obtained using immunofluorescence staining (Fig. 4B, Row 2).

To further understand the role of SOCE in our injury model, we treated the cells with SOCE inhibitors. As shown in Fig. 4C, in the absence of extracellular Ca^2+^, treatment of the control cells with TG induced a significant calcium release, followed by an immediate calcium entry after restoration of extracellular Ca^2+^, indicating the existence of SOCE in HT-22 cells. Cells treated with STIM1 D/E, STIM1-KO, 2-APB, SKF96365, and 5J-4 all had a significant decrease in SOCE-mediated calcium entry, as evidenced by the reduction in calcium entry peak value (Fig. 4C,D) and area under curve (AUC, Fig. 4C,E). However, cells treated with STIM1-shRNA were not as successful at attenuating SOCE-mediated calcium entry as the STIM-KO and SOCE inhibitors. No significant change of TG-induced calcium release was observed among all groups (Fig. 4C–E).

Finally, we evaluated the neuroprotection of SOCE inhibition against glutamate-induced HT-22 cell injury. After glutamate treatment, decreased cell viability (Fig. 4F) and increased LDH release (Fig. 4G) were observed in cells transfected with vector alone as compared to untreated controls. This effect was significantly attenuated in STIM1-KO cells and cells treated with either 2-APB, or 5J-4; however, cells transfected with STIM1-shRNA and cells treated with SKF-96365 did not demonstrate a significant improvement in either measure. Taken together, these results demonstrate that SOCE might be a key mediator of glutamate-induced HT-22 cell injury.

### Up-regulation of Homer1a reduced TG-induced SOCE-mediated calcium entry

**Figure 5:**
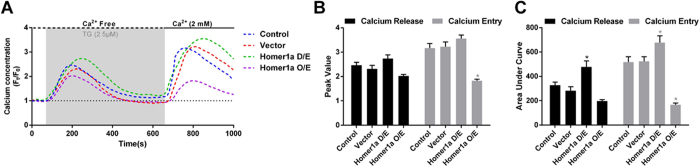
Increased Homer1a protein levels attenuated TG-induced SCOE-mediated calcium entry. After infection with a lentivirus-mediated vector for either Homer1a shRNA or Homer1a O/E for 48 h, HT-22 cells were loaded with Fura-red AM (4 μM) and baseline levels were recorded every 15 s for a total of 100 s. The HT-22 cells were then treated with 2.5 μM TG to release Ca^2+^ from calcium stores (gray zone) in Ca^2+^-free buffer (black dotted line) for 500 s. The buffer was then refreshed with Ca^2+^ buffering (2 mM, black full line). Changes in Ca^2+^ signals were evaluated as time-series curve (**A**) and assessed as peak F/F_0_ (**B**) and area under curve (AUC, **C**). Approximately 40 to 50 cells were analyzed in each experiment. Data are presented as mean ± SEM from four experiments. *p < 0.05 vs. control cells.

After determining the roles of Homer1a up-regulation and SOCE inhibition in glutamate-induced HT-22 cell injury, we further investigated the effect of Homer1a on SOCE. Transfection with the Homer1a shRNA vector significantly increased TG-induced calcium release, as evidenced by an increased AUC (Fig. 5A,C); however, this was not seen with the peak value (Fig. 5A,B). No significant change of calcium release was observed among the other three groups examined, including the control cells, cells + vector or cells transfected with the Homer1a O/E vector (Fig. 5A–C). SOCE-mediated calcium entry was also affected by decreasing Homer1a levels. As shown in Fig. 5A,C, cells transfected with the Homer1a shRNA vector resulted in a slight, but significant, intracellular calcium increase after extracellular calcium was restored; as seen with the calcium release, the peak value of calcium entry was not significantly different from the controls (Fig. 5A,B). In contrast, HT-22 cells treated with the Homer1a O/E vector had a significant decrease both in the peak value (Fig. 5A,B) and the AUC (Fig. 5A,C) as compared with the control cells or cells transfected with vector only. These results demonstrated that an increase in Homer1a levels reduced TG-induced SOCE-mediated calcium entry.

### Up-regulation of Homer1a decreased glutamate-induced STIM1-Orai1 interaction

**Figure 6:**
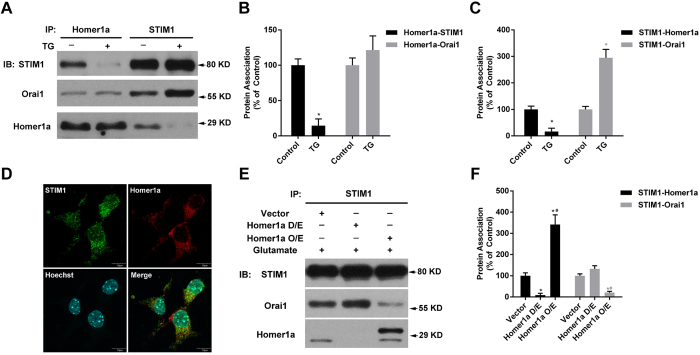
Increased Homer1a protein levels altered STIM1-Orai1 interaction. HT-22 cells were loaded with or without TG (2.5 μM) for 5 min and harvested. Equal amounts of protein were used for Co-IP with anti-STIM1 or anti-Homer1a antibodies, following by western blotting using anti-STIM1, anti-Orai1, or anti-Homer1a antibody (**A**). The relative associations of Homer1a (**B**) or STIM1 (**C**) with each other and Orai1 were quantified as a percentage of the optical density of the control group. HT-22 cells in control group were not subjected to TG. Co-localization of STIM1 and Homer1a was also assessed with immunofluorescence staining (**D)**, STIM1, Green; Homer1a, Red; Nucleus, Blue). Scale bar = 10 μm. After infection with a lentivirus-mediated vector for either Homer1a shRNA or Homer1a O/E for 48h, HT-22 cells were treated with glutamate for 6 h and harvested for Co-IP assay. Equal amounts of protein were used for Co-IP with anti-STIM1 antibody following by western blotting using anti-STIM1, anti-Orai1, or anti-Homer1a antibody (**E**). The relative association of STIM1 (**F**) proteins with Homer1a and Orai1 were quantified as a percentage of the optical density of the vector group. Data are presented as mean ± SEM from four experiments. *p < 0.05 vs. control or vector group, ^#^*p* < 0.05 vs. Homer1a D/E group.

After confirming the inhibition of Homer1a on SOCE-mediated calcium entry, we wanted to further elucidate how Homer1a might affect SOCE machines. In particular, we examined the interaction of Homer1a with both STIM1 and Orai1, two of the main components of SOCE. Co-IP and subsequent western blotting were conducted using normal HT-22 cells and HT-22 cells stimulated with TG (2.5 μM) for 5 min. Under physiological conditions, we detected an interaction of these three proteins (STIM1, Homer1a, and Orai1; Fig. 6A–C, Columns 1 and 3). After treatment with TG, there was a significant decrease in the interaction of Homer1a with STIM1 (Fig. 6A,B, Column 2) and a significant increase of the interaction of STIM1 with Orai1 (Fig. 6A,C, Column 4) as compared with untreated control HT-22 cells. However, significant change of Homer1a-Orai1 interaction was not induced by TG treatment (Fig. 6A, Columns 1 and 2; 5B, Columns 3 and 4). Additionally, immunofluorescence staining showed that partial co-localization of Homer1a and STIM1 also existed under physiological conditions (Fig. 6D). These results suggest that some associations existed among Homer1a, STIM1, and Orai1, either directly or indirectly. Next, we detected the effects of Homer1a D/E or O/E on STIM1-Orai1 association 6 h after glutamate treatment. Cells transfected with the Homer1a shRNA vector slightly, but not significantly, increased STIM1-Orai1 association (Fig. 6E, Column 2; 6F, Column 5). In contrast, cells transfected with the Homer1a O/E vector significantly reduced STIM1-Orai1 associations (Fig. 6E, Column 3; 6F, Column 6). Taken together, these data indicate that Homer1a might play a negative role in regulating STIM1-Orai1 interaction.

## Discussion

The present studies have established a role of Homer1a in oxidative stress injury, negatively affecting STIM1-Orai1 associations, inhibiting SOCE-mediated final-phrase calcium influx, and even alleviating glutamate-induced HT-22 cell death. After treating HT-22 cells with glutamate, the protein levels of Homer1a increased, while the STIM1 localization into puncta within the cell occurred without any apparent change in either STIM1 or Orai1 protein levels. Both increasing Homer1a levels through transfection of the O/E vector and inhibiting SOCE with either SOCE inhibitors or CRISPR/Cas9-mediated STIM1 KO significantly protected HT-22 cells from glutamate-induced oxidative stress injury. Furthermore, TG-induced SOCE-mediated calcium influx and glutamate-provoked STIM1-Orai1 association was attenuated by Homer1a O/E. Therefore, given the role of calcium homeostasis disturbance in oxidative stress injury, therapeutic strategies that can increase Homer1a protein levels and/or inhibit SOCE might serve as potential interventions for neurological diseases in which oxidative stress is involved in either the disease etiology or progression.

Homer1a, as an immediate early gene (IEG), is dynamically regulated by various stimuli, although the mechanisms involved has not been fully elucidated^28^. In line with our previous studies^12^,^27^,^29^, our current study found Homer1a protein levels were significantly increased, peaking 6 h after glutamate treatment. Furthermore, the increased levels of Homer1a attenuated glutamate-induced HT-22 cell death, which agrees with other studies in chronic inflammatory pain, traumatic brain injury, and some neuropsychiatric disorders^12^,^16^,^30^. Together, these studies suggest that the role of Homer1a in neuroprotection is to prevent detrimental or lethal injury to cells. Furthermore, our study found that the neuroprotection of Homer1a was attributed to its ability to inhibit final-phrase calcium overload and reducing mitochondrial oxidative levels, which is in line with our previous study in H_2_O_2_-induced PC12 cell injury^27^. Similar results have also been reported in which the inhibition of calcium influx, by using CoCl_2_ or the use of a Ca^2+^ free buffer alleviated glutamate-induced HT-22 cells death^9^,^10^,^11^. However, the exact mechanism by which Homer1a reduces mitochondrial oxidative levels were not investigated within the scope of this study, and would be warranted in a future study.

SOCE, a crucial regulating mechanism of cellular calcium homeostasis, is dynamically regulated^21^, and previous studies have demonstrated that SOCE is involved in oxidative stress injury^22^. For example, Henke, N. *et al*. found that Orai1 mediates the detrimental Ca^2+^ influx in programmed cell death induced by glutathione depletion or glutamate treatment^8^. Some other investigators found inhibition of STIM1 limits lipopolysaccharide-induced vascular inflammation^25^, alleviated 6-hydroxydopamine-induced oxidative stress^31^, and relieved hydrogen peroxide-induced oxidative injury^26^. In this study, we found changes in the localization of STIM1 after glutamate treatment, with an increase in the presence of STIM1 puncta despite the fact that there was not a detectable change in STIM1 protein levels. This suggests that STIM1 oligomerized after the glutamate treatment. Similar changes in STIM1 protein localization were obtained in COS7 cells after buthionine sulfoximine or H_2_O_2_ treatment^32^. In line with this data, a notable increase of [Ca^2+^]_cyt_ was observed at this timepoint in long-term calcium imaging; however, there was not a significant change in either Orai1 protein levels or its cellular distribution (data not shown). Taken together, these studies suggested that glutamate provoked the redistribution of STIM1, and may cause STIM1-mediated calcium influx. Furthermore, inhibition of SOCE by STIM1 KO, or SOCE inhibitors (2-APB and 5J-4) significantly inhibited TG-induce calcium entry and promoted cell survival against glutamate. However, although treatments of STIM1 D/E and SKF96365 affect TG-induced calcium entry, no significant neuroprotection was observed. This result of STIM1 D/E was partially in agreement with Henke, N. *et al*.’s study^8^; however, our study found that STIM1 D/E was significantly worse at inhibiting TG-induced SOCE, as compared with that of STIM1 KO. One possible reason for these differences seen between the STIM1 D/E and the STIM1 KO might due to residual STIM1 present in cells transfected with the STIM1 D/E vector only, which were sufficient to mediate glutamate-induced calcium influx. In previous studies, SKF96365 was used as a non-special inhibitor of SOCE. Additionally, some off-targets also have been found, like inducing cell apoptosis^33^. Our study in HT22 cells also found that SKF96365 significantly alleviated H_2_O_2_-induced necrosis, but notably increased H_2_O_2_-induced apoptosis (data not shown). This apoptosis might compromise the neuroprotection of SKF96365-mediated SOCE inhibition. However, more efforts should be made in order to clarify the mechanisms involved of SKF96365’s off-target functions.

Homer1b/c has previously been shown to alter both the SOCE machinery and Ca^2+^ release-activated Ca^2+^ channels^18^,^20^,^34^. However, the role of Homer1a has not been clearly defined. Homer1a has been considered as a negative competitor of Homer1b/c due to its lack of a CC domain in C-terminal, which prevents it from self-multimerizing. The present study supported this, as the up-regulation of Homer1a resulted in decreased TG-induced calcium influx and negatively regulated SOCE. Additionally, the associations among STIM1, Homer1a, and Orai1 found in this study are similar to the previously identified associations between STIM1 and Homer1b/c or among STIM1, Homer1b/c, and Orai1^19^,^20^.

Previous studies have established that Homer1 (including Homer1a and Homer1b/c) interacts with other proteins via its EVH1-like domain, which specifically binds to the proline-rich sequences of **PP**XX**F** (where X is any amino acid)^17^, a similar sequences of **P**XX**F** in TRPC (L**P**(P/X)P**F**N) and phosphoinositide 3 kinase enhancer L (**P**KP**F**)^35^,^36^,^37^, or a **LPSSP** sequence^35^. Jardin, I. *et al*. found that TG and thrombin enhanced the Ca^2+^ -dependent interactions among STIM1, Homer1b/c, and Orai1 in human platelets^20^. Likewise, Dionisio, N. *et al*.’s study has indicated that TG induces interactions of Homer1 with STIM1 and the Ca_v_1.2 α1 subunit^19^. In this study, we found that in response to TG-induced calcium store depletion, the association between STIM1 and Orai1 increased, while the association between STIM1 and Homer1a decreased, while no apparent change of Homer1a-Orai1 association was observed. The lack of change in the Homer1a-Orai1 association might be due to the low expression level of Homer1a at resting conditions. However, upon examination of the amino acid sequences of STIM1 and Orai1, while a similar sequence (N**P**AH**F**I) in the C-terminal of STIM1 could be identified, no such sequence could be identified in Orai1. Furthermore, up-regulation of Homer1a significantly reduced glutamate-inducing STIM1-Orai1 association. Therefore, we speculated that Homer1a might dissociate the SOCE complex, and down-regulate SOCE by negatively competing with Homer1b/c.

In conclusion, this study provided experimental evidence that Homer1a is involved in glutamate-induced HT-22 cell death. Overexpression of Homer1a demonstrated a neuroprotective role for this protein in glutamate-induced oxidative stress injury by dissociating the STIM1-Orai1 association and inhibiting glutamate-induced final-phase calcium overload. These results partially uncover the role of Homer1a on SOCE and in oxidative stress injury, and might contribute to the development of new therapeutic strategies. Future studies should determine the exact mechanisms involved in Homer1a-mediated reduction in mitochondrial oxidative level, as well as determine the exact interacting targets and other potential binding partner molecules using either mass spectrometry or point mutations.

## Materials and Methods

### Cell culture

HT-22 cells (Institute of Biochemistry and Cell Biology, SIBS, CAS.) were cultured in DMEM with high glucose (Gibco, Frederick, MD, USA), supplemented with 10% fetal bovine serum (Gibco, Frederick, MD, USA) in a humidified incubator with 5% CO_2_ and 95% air at 37 °C. One day before experiments, HT-22 cells were passaged and seeded in 6-well, 96-well, 60-mm, 100-mm, or confocal culture dishes.

### Western blot

Western blots were performed as previously described^29^. Briefly, proteins were extracted after cell treatments as indicated using lysis buffer supplemented with protease inhibitors (Roche Applied Bioscience, Indianapolis, IN, USA). Protein concentration was quantified with Pierce^®^ BCA Protein Assay Kit (Pierce, Thermo Fisher Scientific Inc., Waltham, MA, USA), and equal amounts of protein (30 μg) were loaded on 8 ~10% SDS-PAGE gels. After electrophoresis, the proteins were transferred to nitrocellulose membranes. The membranes were then blocked with 5% skim milk and incubated at 4 °C overnight with the appropriate primary antibody: STIM1, 1:2,000 (CST, Danvers, MA, USA); β-actin, 1:2,000 (CST, Danvers, MA, USA), Orai1, 1:500 (Novus Biologics, Littleton, CO, USA), or Homer1a, 1:200 (Santa Cruz Biotechnology, Santa Cruz, CA, USA). The blots were then incubated with horseradish peroxidase-conjugated secondary antibodies (1:20000, CST, Danvers, MA, USA), followed by incubation with a chemiluminescent substrate (Thermo Fisher Scientific Inc., Waltham, MA, USA) and detected on film. The optical densities of the protein bands were calculated using an AlphaImager Image Analysis System (Alpha Innotech, East Lyme, CT, USA).

### Plasmids and lentivirus generation

For down-regulation of Homer1a or STIM1, optimal Homer1a shRNA (5′-GCA TGC AGT TAC TGT ATC T-3′), STIM1 shRNA (5′-GCA GTA CTA CAA CAT CAA GAA-3′) or scrambled shRNA (Scr-shRNA, 5′-UUC UCC GAA CGU GUC ACG U-3′) was cloned into the Lenti-pGCL or Lenti-EGFP-pGCL vector. For up-regulation of Homer1a, the cDNA of Homer1a was cloned into the Lenti-pGCL vector. After confirming their sequences with PCR and sequencing analysis, lentivirus preparations were produced (GeneChem Co., Shanghai, China). HT-22 cells were infected by adding lentivirus particles to the cultures at a multiplicity of infection (MOI) of approximately 30. Forty-eight h after infection, HT-22 cells were used for experiments. For knockout of STIM1, three STIM1 single-guide RNAs (sgRNAs) (#1: 5′-TGA GGA TAA GCT TAT CAG CG-3′; #2: 5′-TTA CTG GCC ACG CCA TGC CA-3′; #3: 5′-ACA GTG GCT CAT TAC GTA TG-3′) were cloned into a Lenti-CAS9-sgRNA-puro vector separately. Lentivirus preparations were produced by the Shanghai GeneChem, Co. Ltd, China. After infection with the Lenti-Cas9-STIM1-sgRNA-puro particles (MOI = 10), the HT-22 cells were selected with puromycin (5 μg/L) for seven days and then used for experiments. The knockout efficiency was confirmed by western blot.

### Cell viability assay

The cell viability assays were performed by using the Cell Counting Kit-8 (CCK-8) (Dojindo Lab., Kumamoto, Japan), following the manufacturer’s protocol. Briefly, normal HT-22 cells or lentivirus-infected HT-22 cells were seeded in a 96-well plate with 100 μl culture medium per well (5000 cells/well) and cultured in a humidified incubator for 24 h. Glutamate (5 mM or 10 mM, Ca^2+^-, Mg^2+^-free HBSS; Sigma-Aldrich, St. Louis, MO, USA) was added, and the cells were supplemented with 2-aminoethoxydiphenyl borate (2-APB; 100 μM, DMSO, 0.5‰; Sigma-Aldrich, St. Louis, MO, USA), SKF-96365 (25 μM, ddH_2_O; Sigma-Aldrich, St. Louis, MO, USA), 5 J-4 (20 μM, DMSO, 1‰; TOCRIS, Ellisville, MO, USA) or nothing. Following 24 h of stress onset, 10 μl/well of CCK-8 solution was added to each well. After 4 h incubation in the incubator, the absorbance at 450 nm was measured using a microplate reader (Bio-Rad, Hercules, CA, USA). Cell viability was expressed as a percentage of the control group.

### Lactate dehydrogenase (LDH) leakage assay

To evaluate damaged HT-22 cells in these experiments, the release of LDH was quantitated in the culture medium using the Cytotoxicity Detection Kit^plus^ (Roche Applied Bioscience, Indianapolis, IN, USA) following the manufacturer’s protocol. Following 24 h of the indicated cell treatment, medium was collected for analysis of LDH. After subtracting the background values in the medium, the percentage cytotoxicity was calculated with the following equation: LDH release (% of Max) = 100 × (experimental value − low control)/(high control − low control). Experimental value, LDH values in the experimental groups; Low control, LDH values in the untreated normal cells; High control, the maximum releasable LDH values in the untreated normal cells.

### Calcium imaging and analysis

To evaluate intracellular Ca^2+^ concentration ([Ca^2+^]_cyt_), HT-22 cells were loaded with Fura-Red AM (4 μM; excitation/emission: 494/505–550 nm) (Thermo Fisher Scientific Inc., Waltham, MA, USA) in HBSS Solution (Gibco, Frederick, MD, USA) for 40 min, and then equilibrated for 30 min with refreshed HBSS solution in the dark. The live cell imaging system was heated to 37 °C and ventilated with 6% CO_2_ and 94% air, and the fluorescence changes were determined with a confocal laser scanning microscope (FV10i, Olympus, Tokyo, Japan) in an XYT-plane fashion. In order to detect the SOCE, the buffer was changed with Ca^2+^, Mg^2+^-free HBSS supplemented with 2.5 μM thapsigargin (TG) for 6 min. After this incubation, 2 mM Ca^2+^ was added. For long-term calcium live-cell imaging, HT-22 cells were transfected with the plasmids of pCMV-B-GECO1 (excitation/emission: 370/447 nm) (Addgene, Cambridge, MA, USA) using Lipofectamine LTX-plus (Invitrogen, Carlsbad, CA, USA)^38^. The fluorescence changes were determined at an interval of 1 h in a Multi-XYT-plane fashion and calculated as a ratio of total fluorescence value/total cell number. Cells in which the fluorescence signal quenched were excluded from the analysis. Ca^2+^-insensitive background was subtracted to standardize calcium signal values. The values were then plotted against time and shown as F/F_0_ (F, fluorescence value at any time; F_0_, fluorescence value at the baseline).

### Detection of mitochondrial reactive oxygen level ([ROS]_Mito_)

In order to detect [ROS]_mito_, the HT-22 cells were transfected with the plasmid pMitotimer (Addgene, Cambridge, MA, USA), an engineered reporter gene used to assess mitochondrial content, structure, stress, and damage^39^, using Lipofectamine LTX-plus (Invitrogen, Carlsbad, CA, USA). Images were acquired at the following excitation/emission wavelengths (nm): pMitotimer green (488/518) and red (543/572), and analyzed using Image J (National Institutes of Health, Bethesda, MD, USA). The level of [ROS]_mito_ was reported as the ratio of red/green as previously described^39^.

### Detection of STIM1 and Orai1 distribution

To visualize the distribution of SOCE components within the cells, HT-22 cells were transfected with STIM1-YFP, ER.E2-Crimson or Orai1-YFP (Addgene, Cambridge, MA, USA) using Lipofectamine LTX-plus (Invitrogen, Carlsbad, CA, USA) for 24 h, and then subjected to treatments as described above^40^,^41^,^42^. The localization of STIM1 and ER was captured using a confocal microscope and analyzed using Image J software (National Institutes of Health, Bethesda, MD, USA). The level of STIM1 puncta was calculated as the average/per cell.

### Immunofluorescence staining

After respective treatments, the HT-22 cells were fixed with 4% paraformaldehyde for 10 min, washed with phosphate-buffered saline (PBS), and permeabilized with 0.2% Triton X-100. Next, the HT-22 cells were incubated with primary antibodies overnight at 4 °C as follows: Homer 1a (1:25, Santa Cruz Biotechnology, Santa Cruz, CA, USA) and STIM1 (1:200, CST, Danvers, MA, USA) after blocking with horse serum. Cells were then incubated with Alexa Fluor^®^ 488 or 594-conjugated anti-goat or anti-rabbit secondary antibodies, respectively (1:400, Life Technologies, Carlsbad, CA, USA), for 2 h at room temperature. After washing the cells with PBS three times, the cells were stained with Hoechst stain to visualize the nuclei (Sigma-Aldrich, St. Louis, MO, USA). Cells were imaged using a confocal microscope (FV10i, Olympus, Tokyo, Japan) in XY- or XYZ-plane fashion. Identical light sensitivity and laser power were used among all groups.

### Co-immunoprecipitation (Co-IP)

To evaluate protein interactions, Co-IP experiments were performed using the Pierce™ Crosslink Magnetic IP/Co-IP Kit (Pierce, Thermo Fisher Scientific Inc., Waltham, MA, USA) according to the manufacturer’s instructions. HT-22 cells were cultured in 100-mm dishes, and then harvested in ice-cold lysis/wash buffer supplemented with a proteinase inhibitor cocktail (Roche Applied Bioscience, Indianapolis, IN, USA). The lysate was centrifuge at 13,000 g for 15 min at 4 °C. Protein concentrations in the extracts were determined using a BCA protein assay kit (Pierce, Thermo Fisher Scientific Inc., Waltham, MA, USA). Magnetic beads were crosslinked with non-specific rabbit or goat IgG (2 mg), poly-clonal rabbit anti-STIM1 (1:50; CST, Danvers, MA, USA), or goat anti-Homer1a (1:20; Santa Cruz Biotechnology, Santa Cruz, CA, USA), and then the beads were washed two times with coupling buffer. The protein extracts were combined with the beads and incubated overnight at 4 °C. Following magnetic isolation, the precipitates were washed three times with wash buffer, eluted with the elution buffer, neutralized with neutralization buffer, and prepared for western blotting.

### Statistical analysis

Data are expressed as a mean ± SEM, and analyzed by one-way analysis of variance (ANOVA) followed by Bonferroni’s multiple comparisons among more than two groups or by unpaired t-test comparison between two groups. All statistical analyses were carried out with GraphPad Prism 6 (GraphPad, San Diego, CA, USA). p < 0.05 was considered as the threshold for significant difference.

## Additional Information

**How to cite this article**: Rao, W. *et al*. Homer1a attenuates glutamate-induced oxidative injury in HT-22 cells through regulation of store-operated calcium entry. *Sci. Rep.* **6**, 33975; doi: 10.1038/srep33975 (2016).

## References

[b1] Subramaniam S. R. & Chesselet M. F. Mitochondrial dysfunction and oxidative stress in Parkinson’s disease. Progress in neurobiology 106–107, 17–32, 10.1016/j.pneurobio.2013.04.004 (2013). PMC3742021 23643800

[b2] Pohanka M. Alzheimer s disease and oxidative stress: a review. Current medicinal chemistry 21, 356–364 (2014). 24059239 10.2174/09298673113206660258

[b3] Anand A., Thakur K. & Gupta P. K. ALS and oxidative stress: the neurovascular scenario. Oxidative medicine and cellular longevity 2013, 635831, 10.1155/2013/635831 (2013). 24367722 PMC3866720

[b4] Rodriguez-Rodriguez A., Egea-Guerrero J. J., Murillo-Cabezas F. & Carrillo-Vico A. Oxidative stress in traumatic brain injury. Current medicinal chemistry 21, 1201–1211 (2014). 24350853 10.2174/0929867321666131217153310

[b5] Niizuma K., Endo H. & Chan P. H. Oxidative stress and mitochondrial dysfunction as determinants of ischemic neuronal death and survival. Journal of neurochemistry 109 Suppl 1, 133–138, 10.1111/j.1471-4159.2009.05897.x (2009). 19393019 PMC2679225

[b6] Dixon S. J. . Ferroptosis: an iron-dependent form of nonapoptotic cell death. Cell 149, 1060–1072, 10.1016/j.cell.2012.03.042 (2012). 22632970 PMC3367386

[b7] Kritis A. A., Stamoula E. G., Paniskaki K. A. & Vavilis T. D. Researching glutamate - induced cytotoxicity in different cell lines: a comparative/collective analysis/study. Front Cell Neurosci 9, 91, 10.3389/fncel.2015.00091 (2015). 25852482 PMC4362409

[b8] Henke N. . The plasma membrane channel ORAI1 mediates detrimental calcium influx caused by endogenous oxidative stress. Cell death & disease 4, 10.1038/cddis.2012.216 (2013). PMC3564003 23348584

[b9] Tan S., Sagara Y., Liu Y., Maher P. & Schubert D. The regulation of reactive oxygen species production during programmed cell death. The Journal of cell biology 141, 1423–1432 (1998). 9628898 10.1083/jcb.141.6.1423 PMC2132785

[b10] Ha J. S. & Park S. S. Glutamate-induced oxidative stress, but not cell death, is largely dependent upon extracellular calcium in mouse neuronal HT22 cells. Neuroscience letters 393, 165–169, 10.1016/j.neulet.2005.09.056 (2006). 16229947

[b11] Li Y., Maher P. & Schubert D. Requirement for cGMP in nerve cell death caused by glutathione depletion. The Journal of cell biology 139, 1317–1324 (1997). 9382876 10.1083/jcb.139.5.1317 PMC2140210

[b12] Luo P. . Postsynaptic scaffold protein Homer 1a protects against traumatic brain injury via regulating group I metabotropic glutamate receptors. Cell death & disease 5, e1174, 10.1038/cddis.2014.116 (2014). 24722299 PMC5424101

[b13] Wang Y. . Scaffolding protein Homer1a protects against NMDA-induced neuronal injury. Cell death & disease 6, e1843, 10.1038/cddis.2015.216 (2015). 26247728 PMC4558508

[b14] Hu J. H. . Homeostatic scaling requires group I mGluR activation mediated by Homer1a. Neuron 68, 1128–1142, 10.1016/j.neuron.2010.11.008 (2010). 21172614 PMC3013614

[b15] Ronesi J. A. . Disrupted Homer scaffolds mediate abnormal mGluR5 function in a mouse model of fragile X syndrome. Nature neuroscience 15, 431–440, S431, 10.1038/nn.3033 (2012). PMC3288402 22267161

[b16] Tappe A. . Synaptic scaffolding protein Homer1a protects against chronic inflammatory pain. Nature medicine 12, 677–681, 10.1038/nm1406 (2006). 16715092

[b17] Shiraishi-Yamaguchi Y. & Furuichi T. The Homer family proteins. Genome biology 8, 206, 10.1186/gb-2007-8-2-206 (2007). 17316461 PMC1852408

[b18] Yuan J. P., Lee K. P., Hong J. H. & Muallem S. The closing and opening of TRPC channels by Homer1 and STIM1. Acta physiologica 204, 238–247, 10.1111/j.1748-1716.2011.02319.x (2012). 21518270 PMC3639315

[b19] Dionisio N. . Homer proteins mediate the interaction between STIM1 and Cav1.2 channels. Biochimica et biophysica acta 1853, 1145–1153, 10.1016/j.bbamcr.2015.02.014 (2015). 25712868

[b20] Jardin I., Albarran L., Bermejo N., Salido G. M. & Rosado J. A. Homers regulate calcium entry and aggregation in human platelets: a role for Homers in the association between STIM1 and Orai1. The Biochemical journal 445, 29–38, 10.1042/BJ20120471 (2012). 22506990

[b21] Soboloff J., Rothberg B. S., Madesh M. & Gill D. L. STIM proteins: dynamic calcium signal transducers. Nature reviews. Molecular cell biology 13, 549–565, 10.1038/nrm3414 (2012). 22914293 PMC3458427

[b22] Bogeski I., Kilch T. & Niemeyer B. A. ROS and SOCE: recent advances and controversies in the regulation of STIM and Orai. The Journal of physiology 590, 4193–4200, 10.1113/jphysiol.2012.230565 (2012). 22615429 PMC3473278

[b23] Henke N. . Stromal interaction molecule 1 (STIM1) is involved in the regulation of mitochondrial shape and bioenergetics and plays a role in oxidative stress. The Journal of biological chemistry 287, 42042–42052, 10.1074/jbc.M112.417212 (2012). 23076152 PMC3516750

[b24] Grupe M., Myers G., Penner R. & Fleig A. Activation of store-operated I(CRAC) by hydrogen peroxide. Cell calcium 48, 1–9, 10.1016/j.ceca.2010.05.005 (2010). 20646759 PMC2929316

[b25] Gandhirajan R. K. . Blockade of NOX2 and STIM1 signaling limits lipopolysaccharide-induced vascular inflammation. The Journal of clinical investigation 123, 887–902, 10.1172/JCI65647 (2013). 23348743 PMC3561818

[b26] Rao W. . Blockade of SOCE protects HT22 cells from hydrogen peroxide-induced apoptosis. Biochemical and biophysical research communications 441, 351–356, 10.1016/j.bbrc.2013.10.054 (2013). 24157793

[b27] Luo P. . Protective effect of Homer 1a against hydrogen peroxide-induced oxidative stress in PC12 cells. Free radical research 46, 766–776, 10.3109/10715762.2012.678340 (2012). 22435683

[b28] Serchov T., Heumann R., van Calker D. & Biber K. Signaling pathways regulating Homer1a expression: implications for antidepressant therapy. Biol Chem 397, 207–214, 10.1515/hsz-2015-0267 (2016). 26641965

[b29] Luo P. . Protective effect of Homer 1a on tumor necrosis factor-alpha with cycloheximide-induced apoptosis is mediated by mitogen-activated protein kinase pathways. Apoptosis: an international journal on programmed cell death 17, 975–988, 10.1007/s10495-012-0736-z (2012). 22660975

[b30] Szumlinski K. K., Kalivas P. W. & Worley P. F. Homer proteins: implications for neuropsychiatric disorders. Current opinion in neurobiology 16, 251–257, 10.1016/j.conb.2006.05.002 (2006). 16704932

[b31] Li B., Xiao L., Wang Z. Y. & Zheng P. S. Knockdown of STIM1 inhibits 6-hydroxydopamine-induced oxidative stress through attenuating calcium-dependent ER stress and mitochondrial dysfunction in undifferentiated PC12 cells. Free radical research 48, 758–768, 10.3109/10715762.2014.905687 (2014). 24720513

[b32] Hawkins B. J. . S-glutathionylation activates STIM1 and alters mitochondrial homeostasis. The Journal of cell biology 190, 391–405, 10.1083/jcb.201004152 (2010). 20679432 PMC2922639

[b33] Park E. J. . SK&F 96365 induces apoptosis and autophagy by inhibiting Akt-mTOR signaling in A7r5 cells. Biochimica et biophysica acta 1813, 2157–2164, 10.1016/j.bbamcr.2011.06.021 (2011). 21767581

[b34] Gasperini R., Choi-Lundberg D., Thompson M. J., Mitchell C. B. & Foa L. Homer regulates calcium signalling in growth cone turning. Neural development 4, 29, 10.1186/1749-8104-4-29 (2009). 19650914 PMC2734570

[b35] Yuan J. P. . Homer binds TRPC family channels and is required for gating of TRPC1 by IP3 receptors. Cell 114, 777–789 (2003). 14505576 10.1016/s0092-8674(03)00716-5

[b36] Rong R. . PI3 kinase enhancer-Homer complex couples mGluRI to PI3 kinase, preventing neuronal apoptosis. Nature neuroscience 6, 1153–1161, 10.1038/nn1134 (2003). 14528310

[b37] Barzik M. . The N-terminal domain of Homer/Vesl is a new class II EVH1 domain. Journal of molecular biology 309, 155–169, 10.1006/jmbi.2001.4640 (2001). 11491285

[b38] Zhao Y. . An expanded palette of genetically encoded Ca(2)(+) indicators. Science 333, 1888–1891, 10.1126/science.1208592 (2011). 21903779 PMC3560286

[b39] Laker R. C. . A novel MitoTimer reporter gene for mitochondrial content, structure, stress, and damage *in vivo*. The Journal of biological chemistry 289, 12005–12015, 10.1074/jbc.M113.530527 (2014). 24644293 PMC4002107

[b40] Liou J. . STIM is a Ca2+sensor essential for Ca2+-store-depletion-triggered Ca2+influx. Current biology : CB 15, 1235–1241, 10.1016/j.cub.2005.05.055 (2005). 16005298 PMC3186072

[b41] Gwack Y. . Biochemical and functional characterization of Orai proteins. The Journal of biological chemistry 282, 16232–16243, 10.1074/jbc.M609630200 (2007). 17293345

[b42] Strack R. L. . A rapidly maturing far-red derivative of DsRed-Express2 for whole-cell labeling. Biochemistry 48, 8279–8281, 10.1021/bi900870u (2009). 19658435 PMC2861903

